# Silencing of WNK2 is associated with upregulation of MMP2 and JNK in gliomas

**DOI:** 10.18632/oncotarget.2805

**Published:** 2014-12-22

**Authors:** Angela Margarida Costa, Filipe Pinto, Olga Martinho, Maria José Oliveira, Peter Jordan, Rui Manuel Reis

**Affiliations:** ^1^ ICVS-Life and Health Sciences Research Institute, School of Health Sciences, University of Minho, Campus Gualtar, Braga 4710-057, Portugal; ^2^ ICVS/3B's - PT -Government Associate Laboratory, Braga/Guimarães 4710-057, Portugal; ^3^ INEB-Institute of Biomedical Engineering, Porto 4150-180, Portugal; ^4^ Department of Human Genetics, National Institute of Health Doutor Ricardo Jorge, Lisbon 1649-016, Portugal; ^5^ BioFig-Center of Biodiversity, Functional and Integrative Genomics, Lisbon 1649-016, Portugal; ^6^ Molecular Oncology Research Center, Barretos Cancer Hospital, Barretos, SP 14784-400, Brazil

**Keywords:** WNK2, MMP2, JNK, gliomas, invasion

## Abstract

Matrix metalloproteinases (MMPs) are proteolytic enzymes that degrade extracellular matrix (ECM), thus assisting invasion. Upregulation of MMPs, frequently reported in gliomas, is associated with aggressive behavior. WNK2 is a tumor suppressor gene expressed in normal brain, and silenced by promoter methylation in gliomas. Patients without WNK2 exhibited poor prognosis, and its downregulation was associated with increased glioma cell invasion.

Here we showed that MMP2 expression and activity are increased in glioma cell lines that do not express WNK2. Also, WNK2 inhibited JNK, a process associated with decreasing levels of MMP2. Thus, WNK2 promoter methylation and silencing in gliomas is associated with increased JNK activation and MMP2 expression and activity, thus explaining in part tumor cell invasion potential.

## INTRODUCTION

Gliomas are the most common primary brain tumors, and among them, glioblastomas are not only the most frequent, accounting for approximately 50% of all gliomas, but are also the most aggressive subtype, with a mean survival of 16 months [1]. Due to their high infiltrative and invasive capacity into adjacent tissues, these tumors are extremely difficult to be completely resected during surgery, leading to tumor recurrence, and ultimately patient death [2]. Preventing cancer cell invasion could increase survival of gliomas patients, but the mechanisms that govern this process are poorly understood.

To invade, cancer cells must undergo several changes in cell-cell and cell-matrix adhesion, in migration and proteolysis capacities, and ectopic survival [3]. During cancer cell invasion into adjacent healthy brain tissue, cells need to overcome the extracellular matrix barrier, through a process that involves a tightly regulated proteolytic mechanism, in which MMPs play a pivotal role [3]. MMPs are able to degrade all components of the extracellular matrix, eliminating the physical barrier to cell movement, and creating the route to the invading cells [4].

MMPs are a family of zinc-dependent enzymes, which in the majority of cases are generated and secreted as an inactive form, a pro-enzyme, that has to be processed to become active, autocatalytically or by other proteinases [4, 5]. MMPs are tightly regulated at several levels: transcription, compartmentalization, pro-enzyme activation, and by the action of specific inhibitors, such as tissue inhibitors of MMPs (TIMPs) [6]. Although their main function is extracellular matrix degradation, they are also responsible for the modification of cell surface receptors, release of growth factors, and regulation of inflammation and immunity cytokines, cell-cell adhesion molecules, and other proteinases [6–8]. In gliomas, the importance of MMPs for tumor invasion into the surrounding tissues is well documented [9, 10]. However, the molecular mechanisms that modulate MMPs secretion and activation remain to be clarified.

WNK2, a serine/threonine kinase, is a member of the WNK (with no lysine (K)) protein kinase subfamily, predominantly expressed in brain, heart muscle, small intestine, and colon [11–13]. In epithelial cells the silencing of *WNK2* was reported to increase GTP-loading of Rac1, and the consequent Rac1 activation resulted in increased proliferation mediated by MEK and ERK activation [13, 14]. In tumors, WNK2 expression was found downregulated by promoter hypermethylation both in human gliomas [15, 16] as in other tumor types [17–19]. Given the importance of WNK2 in cancer context, it is important to identify its upstream and downstream targets and determine how they influence WNK2 function. In gliomas, our group recently reported that WNK2 downregulation results in increased cell proliferation, tumor growth, cell migration and invasion [16], corroborating other's theory that WNK2 functions as a tumor-suppressor gene [15, 19].

In the present study, we report for the first time that WNK2 is a modulator of MMPs, negatively regulating MMP2 expression and activity, through a mechanism involving inactivation of JNK. We further demonstrate that downregulation of MMP2 by WNK2 is associated with decreased levels of glioma cell invasion.

## RESULTS

### WNK2 protein expression associates with reduced MMP2 expression and activity

**Figure 1:**
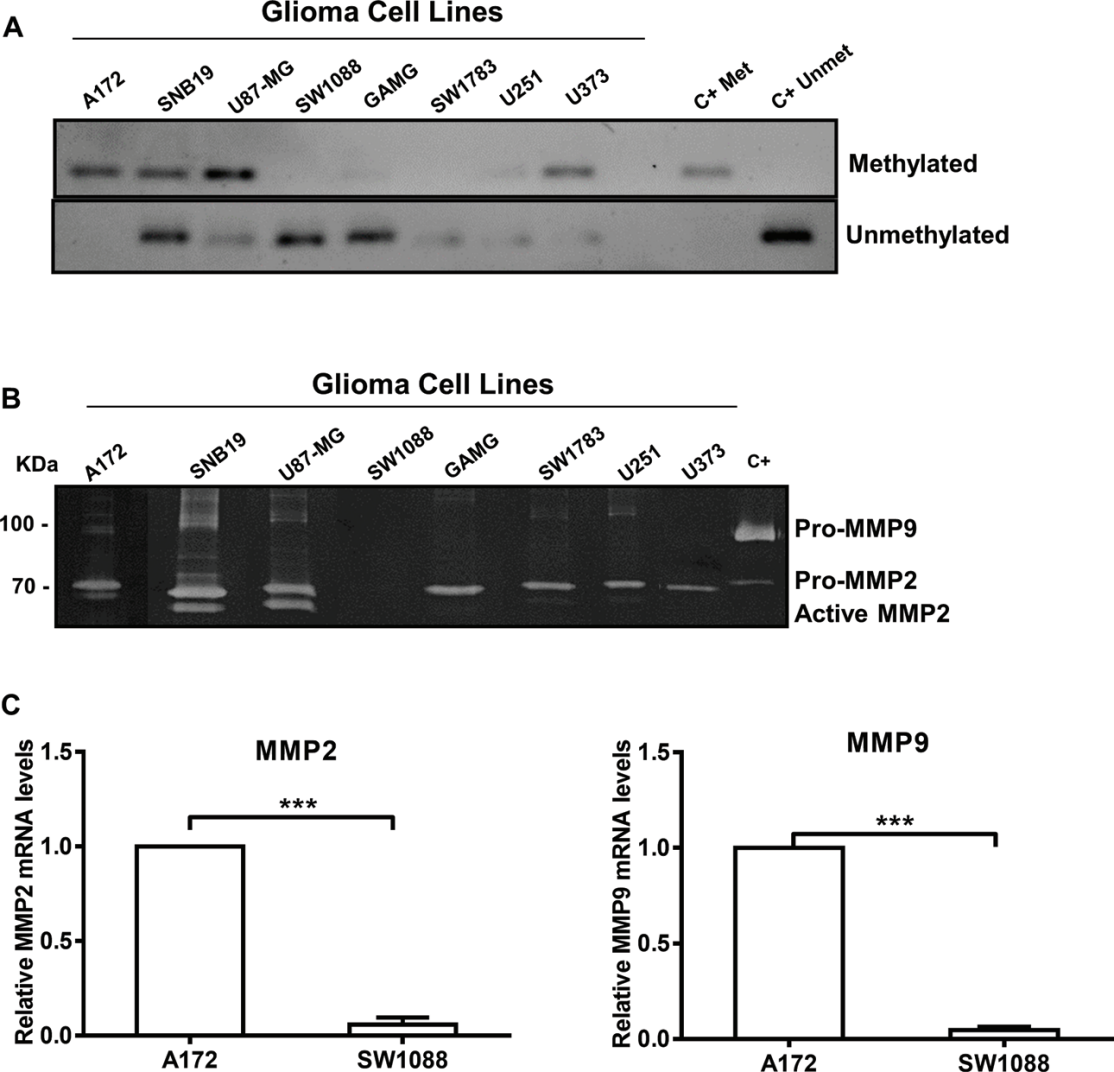
WNK2 protein expression associates with reduced *MMP2* expression and activity **(A)** MSP analysis of the *WNK2* gene promoter in a panel of glioma cell lines. **(B)** The activity of MMP2 and MMP9 was analyzed by gelatin zymography using conditioned medium of glioma cell lines with and without WNK2 expression. C+, positive control for MMP2 and MMP9, conditioned medium of a gastric cell line stimulated with macrophages. **(C)** *MMP2* and *MMP9* mRNA levels were measured by qRT-PCR in A172 and SW1088 cell lines. Relative expression changes in SW1088 are presented as fold increase of MMP/*GAPDH* in comparison to A172 cells. Data on graphs represent the mean values ± standard errors and are representative of, at least, three independent experiments. ***, significantly different from A172 cells (*p* < 0,001).

A high percentage of *WNK2* promoter methylation in gliomas was reported by our and other groups [15, 16], and consequent decrease in the enzyme protein expression was associated with increased levels of glioma cell invasion [16]. We have also previously showed that WNK2 downregulation induces Rac1 activation, leading to increased migration, an important cellular alteration involved in the invasion process [16]. However, the role of WNK2 downregulation in proteolytic events related to glioma cell invasion was not explored. Due to the pivotal role of MMPs in ECM degradation, and consequently to the invasion process, together with the documented association between MMP2 and MMP9 expression and severity of disease in gliomas [20, 21], we interrogated whether the *WNK2* methylation status was associated with MMP2 and MMP9 activity levels in a panel of eight glioma cell lines. For that, the pattern of *WNK2* promoter methylation was analyzed by methylation specific PCR (Figure 1A). Additionally, the levels of MMP2 and MMP9 activity were analyzed by gelatin zymography, using conditioned media of these cells, cultured for 24 hours in serum-free medium. As demonstrated, *WNK2* promoter methylation is associated with increased MMP2 activation levels, and in general also with increased MMP9 protein levels (Figure 1B). Then, two models were chosen to test whether the MMP2 and MMP9 levels were also altered at transcript level: the A172 cell line, with *WNK2* promoter methylation and consequent lack of WNK2 expression, and the SW1088 cell line, with no *WNK2* promoter methylation and endogenous WNK2 protein expression [16]. Cells were cultured in serum-free medium for 24 hours, then RNA was isolated and MMP2 and MMP9 mRNA levels were analyzed by quantitative Real-time PCR (qRT-PCR). As shown in Figure 1C, A172 cells express significantly higher levels of both *MMP2* and *MMP9* mRNA compared to SW1088 cells, (*p* < 0.001), suggesting that WNK2 is involved in the regulation of MMPs transcription.

### WNK2 downregulation leads to an increase in MMP2 RNA levels and activity

**Figure 2:**
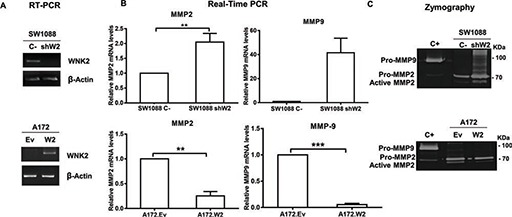
WNK2 downregulation leads to an increase in MMP2 RNA levels and activity **(A)** The expression of WNK2 was analyzed by RT-PCR, using specific primers, and β-actin was used as endogenous control. **(B)** *MMP2* and *MMP9* expression was analyzed by qRT-PCR. MMPs expression levels were normalized to *GAPDH* expression and results are presented as fold differences relative to SW1088 C- or A172.Ev cells. Data correspond to the mean values ± standard errors and are representative of, at least, three independent experiments. **, significantly different from SW1088 C- or A172.Ev cells (*p* < 0.01), ***, significantly different from A172.Ev cells (*p* < 0.001). **(C)** Conditioned medium of indicated cells were run on a gelatin zymogram to detect MMP activity. Proteolytic bands were revealed in white on a Coomassie Blue-stained background.

To define the contribution of WNK2 to *MMP2* and *MMP9* mRNA levels and activity, previously generated stable cell lines [16] were used, namely SW1088 cell line transfected either with a control shRNA (SW1088 C-) or a shRNA directed to *WNK2* (SW1088 shW2), and the A172 cell line transfected either with an empty vector (A172.Ev) or with a *WNK2* expression vector (A172.W2). The levels of *WNK2* expression were confirmed by semiquantitative RT-PCR as shown in Figure 2A. The analysis of *MMP2* and *MMP9* levels by qRT-PCR revealed that the silencing of WNK2 expression results in increased mRNA levels of the analyzed MMPs (Figure 2B). In contrast, ectopic expression of WNK2 caused markedly MMPs' levels decrease (Figure 2B). To confirm if the differences at mRNA levels are also translated into different proteolytic activities, the conditioned media of the four cell lines, cultured for 24 hours in serum-free medium, were analyzed by gelatin zymography. It was found that the absence of WNK2 resulted in increased levels of both the inactive and active form of MMP2, whereas we were no able to find differences regarding MMP9 (Figure 2C). Overall, these results point to an important role of WNK2 as a negative modulator of MMP2 expression and activity.

### WNK2 downregulation is associated with increased SRC and JNK activation levels

**Figure 3:**
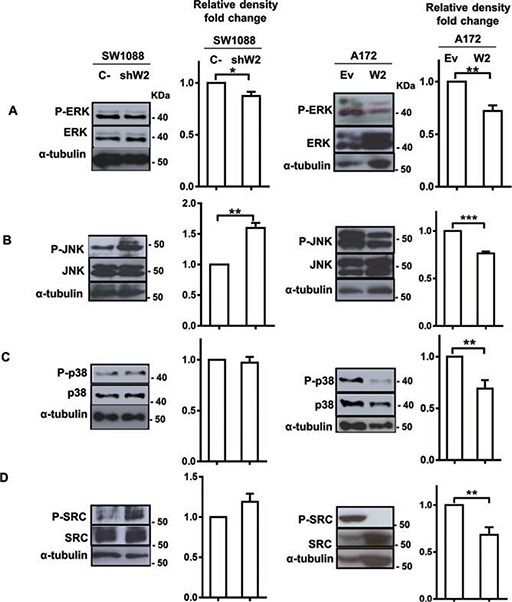
WNK2 downregulation is associated with increased SRC and JNK activation levels Cell lysates were analyzed by Western Blot, immunostained with **(A)** an anti-phospho Thr202/Tyr204 ERK antibody, and re-stained after stripping of the same membrane for total ERK, and α-tubulin **(B)** an anti-phospho Thr183/Tyr185 JNK antibody, and re-stained for total JNK, and α-tubulin, after stripping, **(C)** an anti-phospho Thr180/Tyr182 p38 antibody, and re-stained for total p38, and α-tubulin after stripping of the membrane, **(D)** an anti-phospho Tyr 416-SRC antibody, and re-stained for total SRC, and α-tubulin after stripping. Graphs correspond to the mean values ± standard errors and are representative of, at least, three independent experiments. *, significantly different from SW1088 C- or A172.Ev cells (*p* < 0.05), **, significantly different from SW1088 C- or A172.Ev cells (*p* < 0.01), ***, significantly different from A172.Ev cells (*p* < 0.001).

To elucidate the signaling mechanisms involved in MMP2 upregulation following WNK2 abrogation, we next examined the effect of WNK2 expression in the activation of ERK, JNK, p38, and SRC, pivotal molecules in signaling pathways involved in different MMPs positive regulation, as well as in gliomas' signaling mechanisms [22–29]. For this purpose SW1088 C-, SW1088 shW2, A172.Ev, and A172.W2 cell lines were left three hours in serum-free medium, then total lysates were prepared and the levels of activated phosphorylated ERK, JNK, p38, and SRC analyzed by Western Blot using specific antibodies. WNK2 downregulation in SW1088 cells led to increased levels of JNK and SRC activation, and the opposite is observed when WNK2 is re-expressed in the A172 cell line with no endogenous WNK2 expression (Figure 3B and 3D). In contrast, WNK2 was not associated with ERK and p38 activation in SW1088 cells, although some decrease in the activation levels of ERK and p38 was observed after WNK2 overexpression in A172 cells (Figure 3A and 3C). Given these results, our further studies focused on JNK and SRC modulation by WNK2.

### WNK2 modulates MMP2 expression by negative regulation of JNK

**Figure 4:**
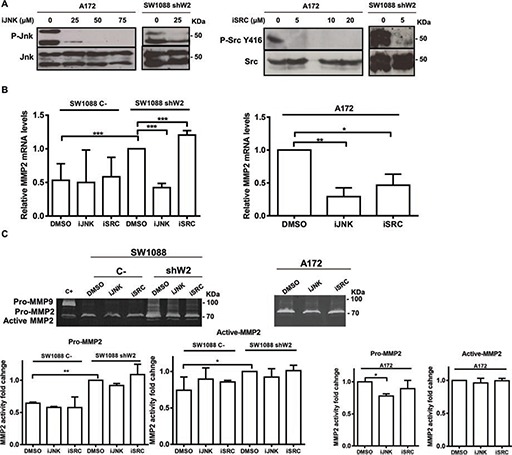
WNK2 modulates MMP2 expression by negative regulation of JNK **(A)** A172 and SW1088 shW2 cells were treated for 3 hours with dimethyl sulfoxide vehicle (DMSO) (0), with a chemical inhibitor of JNK (SP600125; iJNK), or a chemical inhibitor of SRC (Saracatinib; iSRC). After treatment the cell lysates were analyzed by Western Blot, immunostained with anti-phospho Thr183/Tyr185 JNK or anti-phospho Tyr 416-SRC antibodies, and after stripping, the same membrane was immunostained for total JNK, and total SRC respectively. **(B)** The cells were treated for 24 hours with DMSO or the chemicals inhibitors of JNK (SP600125; iJNK) and SRC (Saracatinib; iSRC). *MMP2* mRNA levels were measured by qRT-PCR. Relative expression changes are presented as fold increase of *MMP2*/*GAPDH* in comparison with A172 or SW1088 shW2 treated with DMSO. Data on graphs represent the mean values ±. standard errors and are representative of, at least, three independent experiments. *, significantly different from A172 or SW1088 shW2 cells treated with DMSO (*p* < 0.05), **, significantly different from A172 cells treated with DMSO (*p* < 0.01), ***, significantly different from SW1088 shW2 cells treated with DMSO (*p* < 0.001). **(C)** The cells were treated for 24 hours with DMSO or the chemicals inhibitors of JNK (SP600125; iJNK) and SRC (Saracatinib; iSRC). After the treatment the conditioned medium was collected and analyzed by gelatin zymograms to detect MMP activity. Proteolytic bands were revealed in white on a Coomassie Blue-stained background.

To confirm JNK and SRC as mediators of WNK2 modulation of MMP2, their kinase activity was inhibited and the expression and activity levels of MMP2 were assessed in cells with and without WNK2. For this purpose, parental A172 cells were treated with chemical inhibitors of JNK (SP600125) and SRC (Saracatinib), or with the vehicle (DMSO) alone, at different concentrations, to choose the optimal concentration at which JNK and SRC were inactivated without affecting cell viability (Figure 4A). The viability of cells treated with the chosen concentration of each inhibitor was measured by MTS assay, and showed no significant difference when compared with treatment with DMSO alone (data not shown). The minimal effective concentration was then also confirmed in SW1088 shW2 cells. In order to determine the inhibitor effects on *MMP2* expression by qRT-PCR, and MMP2 activity by gelatin zymography, SW1088 sh C-, SW1088 shW2, and A172 were treated with JNK and SRC inhibitors or DMSO, for 24 hours, then conditioned media were used for zymography, and the cells for RNA extraction. The results showed that in SW1088 C- cells the treatment with JNK and SRC chemical inhibitors did not alter the levels of *MMP2* expression, while in *WNK2*-silenced SW1088 cells the JNK inhibition resulted in reduced *MMP2* mRNA, to levels comparable with control SW1088 cells transfected with a non-silencing shRNA (Figure 4B). The same reduction in *MMP2* levels after JNK inhibition was also observed in parental A172, with no endogenous *WNK2* expression (Figures 4B). In both cells lines with no *WNK2* expression, this *MMP2* reduction also resulted in the decrease of secreted MMP2 levels (Figure 4C). In contrast, the inhibition of SRC resulted in slightly enhanced expression of *MMP2* in SW1088 shW2, and in a decrease of *MMP2* expression in A172 cells, suggesting that the MMP2 regulation by WNK2 is not SRC dependent, but the SRC-dependent *MMP2* expression modulation is potentially dependent of other factors specific of each cell line, independent of *WNK2* expression status (Figure 4B). In both cells lines with no *WNK2* expression the differences observed in *MMP2* expression after SRC inhibition are paralleled by the correspondent alteration in MMP2 secretion (Figure 4C). Taken together, these results suggest that MMP2 expression and activity modulation by WNK2 is partially mediated by JNK.

### MMP2 is involved in glioma cell invasion due to WNK2 downregulation

**Figure 5:**
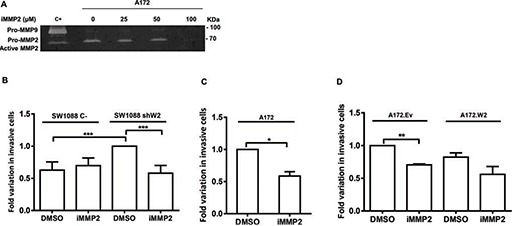
MMP2 is involved in glioma cell invasion due to WNK2 downregulation **(A)** The activity of MMP-2 was analyzed by gelatin zymography using conditioned medium of A172 cells treated for 24 hours with dimethyl sulfoxide vehicle (DMSO) (0), or with a chemical inhibitor of MMP2 activity (ARP-100; iMMP2) at different concentrations. C+, positive control for MMP2 and MMP9, conditioned medium of a gastric cell line stimulated with macrophages. **(B, C, D)** Cells treated with DMSO or with ARP-100 were cultured for 24 hours on Matrigel coated filters. Data on graphs represent the mean values ± standard errors and are representative of, at least, three independent experiments. *, significantly different from A172 treated with DMSO (*p* < 0.05), **, significantly different from A172.Ev cells treated with DMSO (*p* < 0.01), ***, significantly different from SW 1088 C- or SW1088 shW2 cells treated with DMSO (*p* < 0.001).

One of the main MMP2 functions is ECM remodeling leading to cell invasion, namely in gliomas [6, 30], and since our group has previously demonstrated that WNK2 is a negative regulator of glioma cell invasion [16], it was further determined whether MMP2 is involved in invasion of WNK2-negative cells. For this purpose, A172 cells were first treated for 24 hours with different concentrations of a chemical inhibitor of MMP2 activity (ARP 100) or with the vehicle (DMSO) alone, and the conditioned medium collected to perform zymography. Using this strategy, the optimal concentration at which MMP2 activity was inactivated was chosen (Figure 5A). Next, A172, A172.Ev, A172.W2, SW1088 C-, and SW1088 shW2 were treated during the invasion assays either with MMP2 inhibitor or with DMSO alone for 24 hours. The inhibitor has no effect in the number of SW1088 C- invasive cells. SW1088 shW2 cells presented, as expected, increased number of invasive cells compared with SW1088 control cells. Interestingly, the treatment with the MMP2 inhibitor suppressed the invasion induced by the *WNK2* silencing, suggesting the existence of a WNK2-dependent MMP2 activity, which is involved in the glioma invasion (Figure 5B). In the same argument, in A172 cells, with no *WNK2* expression, the MMP2 inhibition was also sufficient to significantly reduce the levels of invading cells (Figure 5C), and the same occurred in A172 cells transfected with an empty vector (Figure 5D). A172 cells stably expressing a *WNK2* vector presented low levels of invasive cells when compared with A172 cells transfected with an empty vector, but in these cells the treatment with the MMP2 inhibitor do not significantly change the number of invasive cells when compared with A172.Ev cells treated only with the MMP2 inhibitor (Figure 5D). These findings demonstrate that WNK2 downregulation stimulate the invasive activity of glioma cells through the action of MMP2, and that MMP2 inhibition, in cells with WNK2, will no further enhance the decrease in the invasion levels, suggesting therefore therapeutic relevance.

### WNK2 negatively regulates IL-6 expression

**Figure 6:**
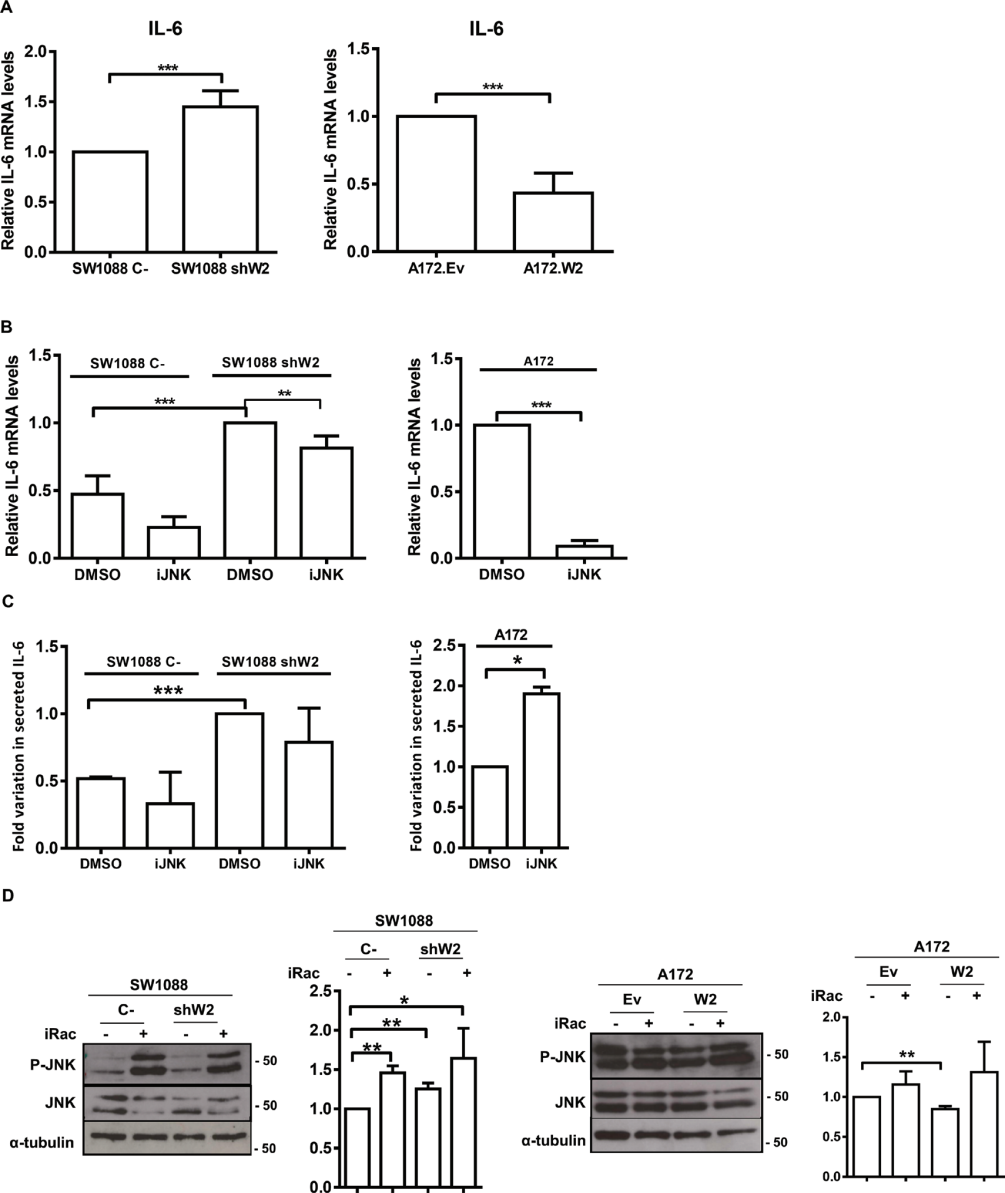
WNK2 negatively regulates IL-6 expression **(A)** *IL-6* expression was analyzed by qRT-PCR. *IL-6* expression levels were normalized to *GAPDH* expression and results are presented as fold differences relative to SW1088 C- or A172.Ev cells. Data correspond to the mean values ± standard errors and are representative of, at least, three independent experiments. ***, significantly different from SW1088 C- or A172.Ev cells (*p* < 0.001). **(B)** The cells were treated for 24 hours with DMSO or the chemical inhibitor of JNK (SP600125; iJNK). *IL-6* mRNA levels were measured by Real-time PCR. Relative expression changes are presented as fold increase of *IL-6*/*GAPDH* in comparison with A172 or SW1088 shW2 treated with DMSO. Data on graphs represent the mean values ±. standard errors and are representative of, at least, three independent experiments. **, significantly different from SW1088 shW2 treated with DMSO (*p* < 0.01), ***, significantly different from SW1088 C-or A172 cells treated with DMSO (*p* < 0.001). **(C)** The cells were treated for 24 hours with DMSO or the chemical inhibitors of JNK (SP600125; iJNK). After the treatment the conditioned medium was collected and analyzed using an IL-6 ELISA assay. IL-6 levels are expressed as fold-increase over the levels in control conditions. Data on graphs represent the mean values ±. standard errors and are representative of, at least, three independent experiments. *, significantly different from A172 cells treated with DMSO (*p* < 0.05), ***, significantly different from SW1088 shW2 cells treated with DMSO (*p* < 0.001). **(D)** Cells were treated for 3 hours with H_2_O (−), or with a chemical inhibitor of Rac1 (NSC23766; iRac) (+). After treatment the cell lysates were analyzed by Western Blot, immunostained with anti-phospho Thr183/Tyr185 JNK antibody, and after stripping, the same membrane was immunostained for total JNK, and α-tubulin. Graphs correspond to the mean values ± standard errors and are representative of, at least, three independent experiments. *, significantly different from SW1088 C- cells (*p* < 0.05), **, significantly different from SW1088 C- or A172.Ev cells (*p* < 0.01).

Gliomas contain high levels of inflammatory cytokines, namely IL-1β, IL-6, and IL-8, which are associated with increase invasion levels [31–33]. Once MMP2 is a regulator of interleukins [6], namely IL-6 [34], and given our previously presented results, we interrogated whether WNK2 modulates IL-6 expression. For that purpose it was used the cell lines SW1088 C-, SW1088 shW2, A172.Ev, and A172.W2. The analysis of *IL-6* levels by qRT-PCR revealed that the silencing of WNK2 expression results in increased mRNA levels of *IL-6* (Figure 6A), and WNK2 re-expression in A172 cells resulted in *IL-6* levels decrease (Figure 6A). To further assess if WNK2-dependent IL-6 modulation occurs in a JNK-dependent manner, SW1088 C-, SW1088 shW2, and A172 cells were treated either with JNK inhibitor (SP600125) or DMSO alone for 24 hours, and the cells used for RNA extraction, and qRT-PCR analysis. The results showed that when JNK activity was inhibited in cells with no *WNK2* expression, the levels of *IL-6* decreased compared with cells treated with DMSO alone (Figure 6B). Taken together, these results showed that WNK2 depletion results in increased mRNA levels of *IL-6*, through a JNK-dependent mechanism.

To further confirm if these results are translated into different IL-6 secretion levels, the conditioned medium of SW1088 C-, SW1088 shW2, and A172, treated or not with the JNK inhibitor for 24 hours in serum-free medium, were analyzed by ELISA. Results confirmed that cells without *WNK2* expression presented higher levels of secreted IL-6, as already observed at the mRNA level (Figure 6C). Regarding JNK modulation of IL-6 secretion, we found that in SW1088 C- and SW1088 shW2 cells the JNK inhibition resulted in a decrease in the IL-6 secreted levels, although in a non-significant manner (Figure 6C). In contrast, the JNK inhibition in A172 cells resulted in increased secreted IL-6 levels, leading us to suggest that *IL-6* increased mRNA expression after WNK2 downregulation is mediated by JNK kinase, although this kinase is not involved in the processes associated with IL-6 secretion (Figure 6C). Since it is known that Rac1 is activated when WNK2 is downregulated [16], and it is described that a Rac/JNK signaling pathway stimulates IL-6 production [35], we tested whether in our model Rac1 is signaling upstream JNK in cells without WNK2 expression. For that purpose, SW1088 C-, SW1088 shW2, A172.Ev, and A172.W2 cells were treated with a Rac1 chemical inhibitor (NSC23766), as previous described [36], and the levels of JNK activation were analyzed by western blot. We found that in every cell line the Rac1 inhibition resulted in higher levels of activated JNK comparing with non-treated cells (Figure 6D), indicating that Rac1 is not signaling through JNK.

### *In silico* validation of inverse correlation of WNK2 with MMP2, MMP9, and IL6 in gliomas

**Figure 7:**
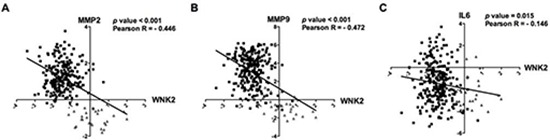
*In silico* validation of inverse correlation of WNK2 with MMP2, MMP9, and IL-6 in gliomas Microarray expression profiles of *WNK2*, *MMP2*, *MMP9* and *IL6* for glioma patients. *In silico* analysis indicates an inverse correlation between WNK2 expression and *MMP2* **(A)** *MMP9* **(B)** and *IL-6* **(C)** expression in glioma patients. Black squares indicate high-grade gliomas, and grey triangles low-grade gliomas.

Since we found that the WNK2 expression status is associated with MMP2, MMP9 and IL6 expression (both mRNA and protein levels, Figures 2 and 6), we further validated and extended our results in human samples using an *in silico* approach. Using the TCGA database (http://www.tcga-data.nci.nih.dov), the expression of WNK2, *MMP2*, *MMP9* and *IL6* genes was evaluated in a cohort of 275 glioma patients. Since the number of GBMs with high *WNK2* expression is very limited (only 4 of 248 cases with log2 median-intensity value > 0), we grouped high and low-grade gliomas in order to assess the correlation of *WNK2* levels with MMPs and *IL6* expression. We found a strong inverse correlation between *WNK2* expression and *MMP2* (*p* < 0.001; Pearson *R* = −0.446) (Figure 7A) and *MMP9* (*p* < 0.001; Pearson *R* = −0.472) levels (Figure 7B), and a low/moderated inverse correlation with *IL6* expression (*p* < 0.015; Pearson *R* = −0.146) (Figure 7C). These results corroborate our *in vitro* findings showing that *WNK2* is a new negative regulator of *MMP2*, *MMP9* and *IL6* expression in gliomas.

## DISCUSSION

Invasion, a hallmark of cancer, is a complex process that involves several cellular modifications such as alterations in cell-cell and cell-matrix adhesion, acquisition of migration capacity and induction of proteolytic activity. The understanding of the molecular mechanisms that govern these cellular alterations is of crucial importance in the attempt of finding effective treatments of cancer. In the case of gliomas, the understanding of the invasive mechanisms gains an extra importance due to their highly invasion capacity, that impairs their complete resection during surgery, significantly reducing the effectiveness of current treatments, and increasing the chances of recurrence [2].

In a previous work, we showed that WNK2 was implicated as a negative regulator of invasion [16]. WNK2 downregulation was associated with an increase in Rac1 activity, a regulator of actin polymerization, which is involved in the establishment of lamellipodia and membrane ruffles, necessary for cell migration [37, 38]. The understanding of the cellular alterations associated with WNK2 downregulation are of great importance given the fact that a high percentage of gliomas present *WNK2* promoter methylation, compared with normal brain tissue [15, 16].

With this work, it was aimed to study whether WNK2 downregulation is associated with increased proteolysis mediated by MMPs, given their central role in extracellular matrix degradation, and consequently, invasion. Our data indicate for the first time that WNK2 downregulation is associated with increased mRNA levels of *MMP2* and *MMP9*, and increased activity levels of MMP2. These data have high relevance since the role of MMP2 in glioma pathogenesis and the association between MMP2 expression, cell invasion and disease severity is well-known [20, 21, 29, 39, 40]. The fact that the upregulation of MMP9 after WNK2 silencing is not associated with increased activity, may suggest that the post-translational activators of MMP2 and MMP9 are different and the activators of MMP9 are not affected by WNK2. By other hand, could be the case that MMP9 alterations occur later in time, once MMP2 is a regulator of other MMPs and have a different activation kinetic.

Having established that *WNK2* depletion is associated with an increase in the MMP2 expression and activity levels, and in the attempt to elucidate the intracellular signaling pathways that are involved in this process, we found that WNK2 negatively regulates JNK and SRC activation, and have no effect neither on ERK nor on p38 activation. It is of note that in case of JNK and ERK, our findings are not concordant with those previously reported in HeLa cervical cancer cells, a phenomena that may be associated with tissue specificities [13]. Interestingly, it was previously reported in gliomas that JNK may function as a positive regulator of MMP2 activity, not by interfering with the MMP expression but by the regulation of TIMP2 expression, an activator of the pro-MMP2 [41]. This study was performed in a rat glioma cell line in which the *WNK2* methylation status was not tested, and it cannot be excluded that the downregulation of WNK2 may activate alternative signaling pathways involving JNK kinase. Regarding SRC, to the best of our knowledge, it was the first time that a relation between this kinase and *WNK2* status was analyzed. Despite the fact that we cannot conclude any association between the levels of SRC activation and MMP2 expression and activity, it is unquestionable that WNK2 modulates SRC activation. This finding is of particular interest because of SRC's role in *in vivo* tumor growth and the promotion of glioma infiltration [42, 43]. The fact that SRC is associated with MMP2 regulation only in A172, and not in SW1088, could be occurring due to cell specific mutations in other genes, not related with WNK2 that can differently regulate SRC-dependent signaling pathways.

MMPs are involved in the regulation of several cellular processes beyond the ECM degradation, such as modification of cell surface receptors, release of growth factors, and regulation of inflammation and immunity cytokines, cell-cell adhesion molecules, and other proteinases [6–8]. To assess whether the alterations in MMP2 related to the WNK2 status were involved in the invasive process, glioma cells with or without WNK2 were treated with a chemical inhibitor of MMP2 activity. The results obtained after performing matrigel invasion assays indicate that MMP2 is necessary for glioma cell invasion, and that the WNK2 presence is sufficient to suppress the invasive phenotype. In cases where cells present WNK2 expression further inhibition of MMP2 activity have not any additional benefit to the invasion impairment. Judging from these results, we can suggest that the reduction of MMP2 expression levels, mediated by JNK inhibition, is sufficient and strong enough to alter the invasive behavior of the cell, impairing the infiltration of glioma.

Previous studies have shown the importance of IL-6 chemokine in the invasive behavior of gliomas [31, 32] and its association with MMP2 [33, 34]. Additionally, Rac/JNK signaling pathway was described to stimulate IL-6 production in mast cells [35]. Therefore, we decided to address the issue of IL-6 regulation mediated by WNK2. We reported for the first time that WNK2 negatively regulates *IL-6* mRNA expression, through JNK activity inhibition, once again pointing to the advantage of JNK inhibition in the disease context. These correlations were further corroborated by *in silico* analysis gathered by the TCGA dataset consisting of 275 gliomas patient.

Our group previously showed that Rac1 is upregulated following WNK2 silencing in gliomas [16]. Moreover, Rac1 is a known upstream regulator of JNK activity [44, 45], and both Rac1 and JNK are reported to be involved in MMP2 upregulation [46, 47]. Thus, we decided to evaluate whether Rac1 is signaling through JNK when WNK2 is downregulated. Our results showed that the Rac1 inhibition did not result in decrease JNK activation levels - as it was expected if Rac1 was signaling through JNK - but instead, we observed an increase in the JNK activation levels, eventually indicating a potential compensation mechanism, a frequent event in other signaling pathways [48].

Additionally, the present study can have potential therapeutic impact. It is known that MMP inhibitors are ineffective as therapeutic agents [49], therefore, targeting of molecules that function upstream the MMP expression and activation, would be of great value. In this line, our results point to the fact that either WNK2 re-expression or JNK inhibition may improve therapeutic modalities in the treatment of gliomas. Importantly, it has been reported that JNK inhibition enhances temozolomide cytotoxicity in human gliomas [50]. If we assume that JNK is a general MMP2 inhibitor in gliomas, even patients with normal WNK2 expression treated with a JNK inhibitor would better benefit from the treatment, both because of the invasion inhibition, and by the enhancement of temozolomide cytotoxicity.

In conclusion, the results reported in this study indicate, for the first time, that WNK2 silencing results in JNK activation, which in turn positively regulates IL-6 and MMP2 expression and MMP2 activity, leading to increased levels of cell invasion. These *in vitro* findings may contribute to our understanding of the mechanisms that lead to glioma cell invasion in cases where *WNK2* is silenced by promoter hypermethylation, and provide a novel therapeutic strategy in glioma treatment.

## MATERIALS AND METHODS

### Cell culture and reagents

The adult glioma cell lines A172, U87-MG, SW1088, and SW1783, (ATCC; Manassas, VA, USA), SNB19, and GAMG (DSMZ; Braunschweig, Germany), U251, and U373 (kindly provided by Costello Laboratory [51]) were maintained in DMEM (Gibco; Grand Island, NY, USA) with 10% fetal bovine serum (FBS) (Gibco), 200 μg/ml streptomycin, and 200 IU/ml penicillin (Gibco) at 37°C, under a humidified atmosphere containing 5% CO2. A172.Ev, A172.W2, SW1088 C-, and SW1088 shW2 were generated and maintained as previously described [16].

### Methylation specific PCR

Genomic DNA from glioma cell lines was isolated using TRIzol^®^ Reagent (Invitrogen; Barcelona, Spain). 300 ng of DNA were bisulphite treated using EZ DNA Methylation-Gold^TM^ kit (Zymo Research Corporation, Irvine, CA, USA), and PCR performed using the specific primers described before [16]. Cp Genome^TM^ Universal Methylated DNA (Millipore; Billerica, MA, USA) was used as methylated control and blood DNA of a young healthy individual was used as unmethylated control.

### Preparation of cell lysates

Cells were lysed in RIPA lysis buffer (50 mM Tris-HCl, 1% NP-40, 150 mM NaCl, 2 mM EDTA, pH 7.5) with protease inhibitors (Complete Mini protease inhibitor cocktail tablets, Roche; Mannheim, Germany) and phosphatase inhibitors (1 mM sodium vanadate, 0.1 mM NaF, 2.5 mg/mL sodium pyrophosphate). 25 μg of protein were used for total lysates analysis. Proteins were separated by SDS-PAGE.

### Immunoblot analysis

Proteins were transferred onto Hybond nitrocellulose membranes (GE Healthcare; Little Chalfont, Buckinghamshire, UK), blocked with 5% non-fat milk in PBS +0.1% Twen-20 (PBS-T) and incubated overnight at 4°C with primary antibody. The following antibodies were used: anti-phospho ERK (P-p44/42 MAPK T202/Y204), anti-phospho JNK (P-SAPK/JNK T183/Y185), anti-phospho p38 (T180/Y182), anti-phospho SRC (Y416), anti-ERK, anti-JNK, anti-p38, anti-SRC (all from Cell Signaling Technology; Danvers, MA, USA), and anti-α-tubulin (Sigma-Aldrich; Madrid, Spain). HRP-conjugated goat anti-mouse and goat anti-rabbit (Santa Cruz Biotechnology; Santa Cruz, CA, USA) were used as secondary antibodies. Subsequently ECL detection (SuperSignal^®^ West Femto, Thermo Scientific; Waltham, MA, USA) was performed. Band intensity was quantified using Image J software.

### Preparation of conditioned medium

Conditioned medium was collected after cell maintenance in a serum-free medium for 24 hours, centrifuged at 13000 rpm for 5 min and filtered through 0.2 μm pore size filters (Sterile Acrodisc^®^, Pall Corporation; Port Washington, NY, USA). To assess MMPs activity, conditioned medium was concentrated with acetone.

### Zymography

MMP2 and MMP9 activity was assessed using concentrated protein from the conditioned medium. In each condition 5 μg of protein were loaded on a 10% SDS-PAGE, containing 1 mg/mL gelatin as substrate. Zymograms were run in a Tris/glycine SDS running buffer under nondenaturing conditions. After electrophoresis, gels were washed twice with 2% Triton X-100, to remove SDS remnants. Zymograms were subsequently incubated for 16 hours at 37°C in a MMP substrate buffer (50 mM Tris-HCl, 10 mM CaCl_2_, pH 7.5). Proteolytic activity was visualized as the presence of clear bands against a blue background after Coomassie Blue gel staining.

### Quantification of mRNA by quantitative Real-Time PCR and semiquantitative RT-PCR

Total RNA was extracted using TRIzol^®^ Reagent (Invitrogen). One μg of RNA was used for cDNA synthesis, using the High-Capacity cDNA Reverse Transcription Kit (Applied Biosystems; Branchburg, NJ, USA). Semiquantitative RT-PCR to *WNK2* was performed using the primers Fw (5′-GTGCACGATCCTGAAATCAA-3′) and Rv (5′- CAGTTTCTTGGGGTCTTCCA-3′) (exon 6), and *β-actin* was used as housekeeping gene with the primers Fw (5′- GGACTTCGAGCAAGAGATGG-3′) (exon 3), and Rv (5′- AGCACTGTGTTGGCGTACAG-3′) (exon 4), and Platinum^®^ *Taq* DNA Polymerase (Invitrogen). Real-time PCR to *MMPs* was performed using TaqMan Mix (Applied Biosystems) and the *MMP2* (Hs01548727_m1) and *MMP9* TaqMan probes (Hs00234579_m1) (Applied Biosystems). Relative *MMP* expression was normalized to levels of *GAPDH* (Human GAPDH Endogenous Control (FAM™ Dye/MGB Probe, Non-Primer Limited), (Applied Biosystems). PCR was performed in a 7500 Real Time PCR System (Applied Biosystems).

Real-time PCR to *IL-6* was performed using the SsoFast^TM^ EvaGreen^®^ Supermix (BioRad; Hercules, CA, USA), according to the manufactures' instructions, using the *IL-6* primers Fw (5′-AAAGAGGCACTGGCAGAAAA-3′) and Rv (5′- CAGGGGTGGTTATTGCATCT-3′) (exon 2), and *β-actin* was used as housekeeping gene with the primers Fw (5′- GGACTTCGAGCAAGAGATGG-3′) (exon 3), and Rv (5′- AGCACTGTGTTGGCGTACAG-3′) (exon 4). Relative *IL-6* expression was normalized to levels of *β-actin.* PCR was performed in a Bio-Rad CFX96TM Real Time PCR System (Bio-Rad).

### Cell treatment

Pharmacological inhibitors SP600125 and ARP 100 were obtained from Cayman Chemicals (Ann Arbor, MI, USA), Saracatinib from Selleckchem (Houston, TX, USA), and NSC23766 from Calbiochem (San Diego, CA, USA). The concentrations of inhibitors used were 25 μM for SP600125, 5 μM for Saracatinib, and 100 μM for ARP 100 and NSC23766. Cells were treated for 3 hours in serum-free medium for total cell lysates, or for 24 hours, for conditioned medium, RNA, and invasion assays.

### Cell viability assay

Cells were plated in a 96-wells plate at a density of 3 × 10^3^ per well, and allowed to adhere overnight. Cells were treated with chemical inhibitors or with DMSO, as a negative control, in DMEM supplemented with 0.5% FBS for 24 hours. After this period, cell viability was quantified using CellTiter 96 Aqueous Cell Proliferation Assay (MTS) (Promega; Madison, WI, USA). The results were expressed as mean viable cells in relation to DMSO alone, considered as 100% viable.

### Invasion assays

Matrigel-coated 24-well invasion inserts of 8 μm pore-size filters (BD Biosciences; Madrid, Spain) were incubated one hour at 37°C with antibiotic-free medium. After this period, 5 × 10^4^ cells, in antibiotic-free medium, either with the MMP2 chemical inhibitor (ARP 100), or with DMSO, as negative control, were incubated on top of the filters for 24 hours at 37°C. Filters were washed with PBS and fixed in cold methanol. Invasive cells were stained and mounted in Vectashield with DAPI (Vector Laboratories; Burlingame, CA, USA), and visualized through an Olympus BX61 fluorescence microscope (Olympus, Hamburg, Germany). Invasive cells were scored in at least 20 microscopic fields (20X objective).

### IL-6 ELISA assay

Conditioned media from cells was collected after 24 hours treatment with the JNK inhibitor or DMSO in serum free medium. Quantification of secreted IL-6 was assessed by an IL-6 ELISA kit (LEGEND MAX^TM^ Human IL-6, Biolegend, San Diego, CA, USA), according to the manufacturer's instructions. IL-6 levels were expressed as fold-increase over the levels in control conditions.

### *In silico*, microarray expression analysis

Microarray expression data was downloaded for 275 glioma patients (248 high grade and 27 low grade gliomas) with expression available for WNK2, MMP2, MMP9, and IL6 from The Cancer Genome Atlas (TCGA) database (http://www.tcga-data.nci.nih.dov).

### Statistical analysis

Data were analyzed with Student's t-test and expressed as mean values of at least three independent experiments ± standard errors. Pearson test was used to evaluate the correlation between gene expression profiles from TCGA database. Differences were considered significant at *p < 0.05, **p < 0.01, and ***p < 0.001.
